# Experimental linear-optics simulation of ground-state of an Ising spin chain

**DOI:** 10.1038/s41598-017-02571-w

**Published:** 2017-05-19

**Authors:** Peng Xue, Xian Zhan, Zhihao Bian

**Affiliations:** 1 Department of Physics, Southeast University, Nanjing, 211189 China; 2 State Key Laboratory of Precision Spectroscopy, East China Normal University, Shanghai, 200062 China

## Abstract

We experimentally demonstrate a photonic quantum simulator: by using a two-spin Ising chain (an isolated dimer) as an example, we encode the wavefunction of the ground state with a pair of entangled photons. The effect of magnetic fields, leading to a critical modification of the correlation between two spins, can be simulated by just local operations. With the ratio of simulated magnetic fields and coupling strength increasing, the ground state of the system changes from a product state to an entangled state and back to another product state. The simulated ground states can be distinguished and the transformations between them can be observed by measuring correlations between photons. This simulation of the Ising model with linear quantum optics opens the door to the future studies which connect quantum information and condensed matter physics.

## Introduction

Quantum simulator^1–9^ provides a platform that allows to reproduce the behaviour of different complex system. Compared to quantum computation, quantum simulations are conjectured to be less demanding by being less stringent on accurate gate operations and error corrections. Due to the advantages, quantum simulation has been paid attention recently and led to many theoretical proposals^10–13^. Various quantum simulators based on different physical platform are being constructed, such as atoms in optical lattice^14–17^, trapped ions^18–20^, nuclear magnetic resonance^21^, superconducting circuits^22^, and photons^23^.

Recently, much attention has been attracted by quantum simulation of spin models which shed light on a variety of open problems such as quantum phase transition^24, 25^. Quantum entanglement^21, 26–29^ of ground states of spin models is one of the aspects. It is important to simulate the ground state of spin models^20, 30–34^ that are expected to exhibit highly nonclassical properties.

We experimentally simulate the ground state of an Ising spin chain and characterize the structural changes. That explains the nature of the ground state changes discontinuously. We observe a transformation from a simple product state to a strongly entangled state of an Ising spin chain with different parameters of the system which can be varied by only local operations. We study non-locality and two-spin correlation in an Ising chain. Compared to the previous experiment in ref. 30, we focus on simulation of the ground state transformation. The ground state is transmitted from the ferromagnetically ordered high-field states to the entangled anti-ferromagnetic low-field states and the transformation can be monitored by the changes of the ground state of the spin chain as the model approaches its critical point.

## Results

### Theoretical frame

We consider the Hamiltonian of an Ising spin chain^31^

1 $$\hat{ {\mathcal H} }=\sum _{i}\,\frac{{\omega }_{z}}{2}{\hat{\sigma }}_{z}^{i}+\frac{{\omega }_{x}}{2}{\hat{\sigma }}_{x}^{i}+{\mathscr{J}}{\hat{\sigma }}_{z}^{i}{\hat{\sigma }}_{z}^{i+1},$$

where ${\hat{\sigma }}_{z}^{i}$ (${\hat{\sigma }}_{x}^{i}$) is Pauli operator applied on the *i*th spin, *ω*_*z*_ (*ω*_*x*_) is a magnetic-field strength, ${\mathscr{J}}$ is a strength of dipole-dipole interaction between two nearest-neighbor spins and $|{\omega }_{x}|\ll |{\omega }_{z}|$, $|{\mathscr{J}}|$ is satisfied.

Consider a two-spin system (an isolated dimer) governed only by an Ising interaction (1), the ground state of the system, i.e., the eigenstate with the lowest energy, is two-fold degenerated. Without a transverse magnetic field (i.e., *ω*_*x*_ = 0), the eigenbasis^31^ is spanned with the four states $\{|00\rangle ,|{{\rm{\Psi }}}^{+}\rangle ,|11\rangle ,|{{\rm{\Psi }}}^{-}\rangle \}$, where $|{{\rm{\Psi }}}^{\pm }\rangle =(|01\rangle \pm |10\rangle )/\sqrt{2}$ are maximally entangled states and |0〉 and |1〉 represent the spin down and up states.

There are two blocks of the Hilbert space spanned by the eigenstates of the Hamiltonian (1)^31^ for vanishing transverse field. The triplet states are in one of the blocks and the singlet state is in the other block. The transformations from the triplet states to the singlet state are symmetry forbidden. In the actual experimental realization, we only consider the case in which the initial state is |00〉. Since the transformation from |00〉 to the singlet state is symmetry forbidden, then we can reduce our system of interest to the triplet states. That is because we want to simulate quantum state transportation: with the ratio of magnetic fields and coupling strength changing, the ground state is transmitted from the ferromagnetically ordered high-field states to the entangled anti-ferromagnetic low-field states. The state transportation can only occur in one of the blocks of the Hilbert space spanned by the four eigenstates. Then we choose to simulate the triplet eigenstates and to simulate the transformation within this block. Thus we do not take the singlet eigenstate |Ψ^−^〉 into account.

With longitudinal field *ω*_*z*_ and small transverse field ${\omega }_{x}\ll 1$, the ground states of the Ising spin model^31^ are determined as $|{\psi }_{g}\rangle \approx |00\rangle$ for *β*_*z*_ < −1, $|{\psi }_{g}\rangle \approx |{{\rm{\Psi }}}^{+}\rangle$ for −1 < *β*_*z*_ < 1 and $|{\psi }_{g}\rangle \approx |11\rangle$ for *β*_*z*_ > 1. Here the ratio of magnetic field and coupling strength ${\beta }_{x(z)}={\omega }_{x(z)}/\mathrm{(2}{\mathscr{J}})$ is defined as the dimensionless field strength. The quantum critical points are achieved when *β*_*z*_ = ±1 is satisfied. At the quantum critical points, the ground state of the Ising spin chain changes from the ferromagnetically ordered high-field states (i.e., the separated states |00〉 and |11〉) to the anti-ferromagnetic low-field state (i.e., the entangled state |Ψ^+^〉).

**Figure 1:**
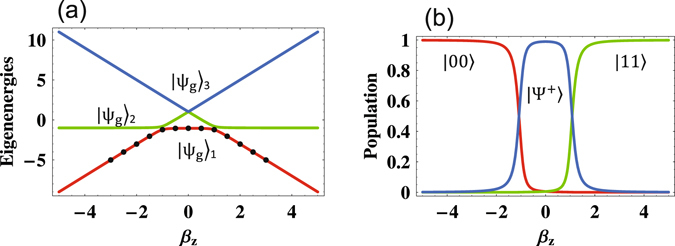
Theoretical predictions. (**a**) Energy spectrum for the two-spin Ising chain (an isolated dimer) as a function of the dimensionless field strength *β*_*z*_ and a fixed *β*_*x*_ = 0.15. We experimentally simulate the ground state |*ψ*_*g*_〉_*i*_ of the Ising spin model with the certain dimensionless field strengths {*β*_*x*_, *β*_*z*_} = {0.15, 0}, {0.15, ±0.5}, {0.15, ±1}, {0.15, ±1.5}, {0.15, ±2}, {0.15, ±2.5}, {0.15, ±3}. (**b**) Populations of |00〉, |11〉 and |Ψ^+^〉 in the ground state |*ψ*_*g*_〉_*i*_ v.s. *β*_*z*_.

The energy spectrum in Fig. 1a shows that the characterization of the ground state of an Ising spin chain^31^ mainly depends on the dimensionless longitudinal field strength *β*_*z*_. That is, with a strong longitudinal magnetic field, the ground state is a product state. whereas it turns to be an maximally entangled state with a weak longitudinal magnetic field. By decreasing the strength of longitudinal magnetic field, the ground state changes from the ferromagnetically ordered high-field states to the entangled anti-ferromagnetic low-field states. The critical point of the ground state transportation is therefore achieved if the strength of magnetic field equals to that of dipole-dipole interaction.

We experimentally demonstrate quantum simulation of the ground state of the two-spin Ising model with certain parameters {*β*_*x*_, *β*_*z*_}. We use the same figures of merit as used in ref. 31 to characterize the property of the ground state, such as the concurrence and two-spin correlation $\langle {\sigma }_{z}^{1}{\sigma }_{z}^{2}\rangle$. Our goal is to simulate the ground state of an Ising spin chain and observe the ground state transformation, i.e. ferromagnetic-to-anti-ferromagnetic transformation, in a real physical system. We quantify the simulated ground state with full quantum state tomography.

### Experimental realization

Linear optics is one of the leading architectures for quantum simulators^27, 28, 30–32^, due to the strong power of control of the wavefunction and the high robust to decoherence and noise. In this paper we realize an optical quantum simulator by encoding the wavefunction of the ground state of a two-qubit Ising spin chain (an isolated dimer) with a pair of polarization-entangled photons which are generated via spontaneous parametric down-conversion (SPDC). The magnetic field, resulting in the possibility to explore the characters of the ground state of the Ising spin chain, is then simulated by variable rotation on the prepared photonic states. Correlations between the two spins can be measured via the coincidence of photon pairs in different basis.

**Figure 2:**
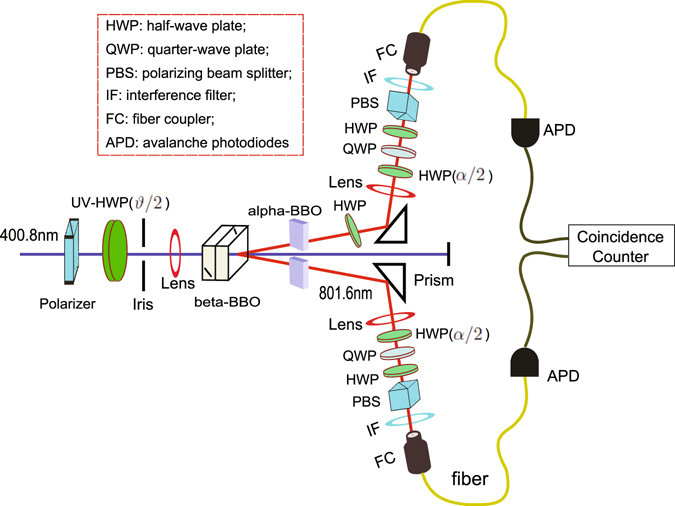
Experimental schematic. A 400.8 nm pump is directed injected through two 0.5 mm-thick *β*-BBO crystals, giving rise to pairs of correlated photons at 801.6 nm. Photon pairs are detected via APDs and fast coincidence electronics. Quartz plates (not shown) and *α*-BBO crystals are used to compensate the birefringence of the *β*-BBO crystals. The following HWP is used to adjust the relative phase of entangled photon pairs. The simulated ground state of the two-spin Ising model changes from a ferromagnetically ordered high-field states to an anti-ferromagnetic low-field states via tuning the setting angles *ϑ*/2 and *α*/2 of the corresponding HWPs. A two-photon tomography of the output allows reconstruction of the simulated ground state.

The experimental setup for realization of photonic quantum simulator is shown in Fig. 2. The photon pairs^35^ at wavelength 801.6 nm are generated in the non-maximally entangled state

2 $$|\varphi \rangle \,=\,\cos \,\vartheta |HH\rangle \,+\,\sin \,\vartheta |VV\rangle $$

via type-I SPDC in two 0.5 mm-thick nonlinear-*β*-barium-borate (BBO) crystals, which are pumped by a CW diode laser (400.8 nm) with 90 mW of power. Here |*H*〉 = |0〉 and |*V*〉 = |1〉 correspond to the horizontal and vertical polarizations of photons, respectively. The parameter *ϑ* is controlled simply by the polarization of the pumping laser which is prepared in the state cos *ϑ*|*H*〉 + sin *ϑ*|*V*〉 and can be tuned by an ultraviolet half-wave plate (HWP) with the setting angle *ϑ*/2 (the angle between optical axis of the HWP and the horizontal direction). In our experiment, we generate entangled photon pairs and inject them in the subsequence logical circuit. In combination with narrowbandwidth filters of 3 nm this procedure yields fidelity for the maximally entangled state (*ϑ* = *π*/4) as high as 98%.

Adjusting the subsequent local operations via a set of HWPs allows us to tune the coefficients of the states of photon pairs. We perform the single-qubit rotation on both photons in a pair with a HWP with the angle between the optical axis and the horizontal direction setting to *α*/2. The matrix for the rotation is

3 $${R}_{{\rm{HWP}}}(\frac{\alpha }{2})=(\begin{array}{cc}\cos \,\alpha  & \sin \,\alpha \\ \sin \,\alpha  & -\cos \,\alpha \end{array}).$$

After the rotations applied, the pairs of photons are prepared in the state

4 $$\begin{array}{rcl}|\varphi ^{\prime} \rangle  & = & {R}_{{\rm{HWP}}}(\frac{\alpha }{2})\otimes {R}_{{\rm{HWP}}}(\frac{\alpha }{2})|\varphi \rangle \\  & = & \frac{1}{\sqrt{{a}^{2}+{b}^{2}+2{c}^{2}}}(a|HH\rangle +b|VV\rangle +\sqrt{2}c|{{\rm{\Psi }}}^{+}\rangle ),\end{array}$$

where

5 $$\alpha =-\,\frac{1}{2}\,\arccos \,\frac{a-b}{\sqrt{2-{(a+b)}^{2}}},\,\vartheta =\arcsin \,\frac{a+b}{\sqrt{2}}-\frac{\pi }{4}.$$

We can simulate the ground state of two-spin Ising model by choosing proper coefficients *a*, *b* and *c*, which can be accomplished by setting the HWPs to the proper angles *ϑ*/2 and *α*/2.

The transverse and longitudinal magnetic fields are then simulated by the rotations of the polarizations of photons. Using this setup we are able to prepare the photon pairs in the eigenstates of the Ising spin model and to observe the ground state transportation by applying the local rotations in each photon via HWPs with the proper setting angles *ϑ*/2 and *α*/2. For any dipole-dipole interaction strength ${\mathscr{J}}$, the strengths of the transverse and longitudinal fields *ω*_*x*_ and *ω*_*z*_, one can find the corresponding coefficients *a*, *b* and *c* in Eq. (4). The coefficients can be tuned by the setting angles of the HWPs *ϑ*/2 and *α*/2 shown in Eq. (5). Output photons are detected using avalanche photo-diodes (APDs, 7 ns time window) with dark-count rate of less than 100 s^−1^ whose coincidence signals, monitored using commercially available counting logic, are used to post-select two single-photon events. We record clicks for 20 s, and the total coincidence counts are ~2000. The rotation on each photon via HWP and quarter-wave plate (QWP), along with polarizing beam splitter (PBS), enables analysis of the polarization corrections in any basis, allowing tomographic reconstruction of the density matrix. In practice, the tomographic measurements are only performed on the optical paths with successful entanglement concentration. We characterize experimental performance via the fidelity of the simulated ground states shown in Table 1

6 $$ {\mathcal F} ={}_{1}\langle {\psi }_{g}|\rho |{\psi }_{g}\rangle _{1},$$

where *ρ* is the reconstructed two-qubit state. To calculate the error bar of the fidelity, the statistics of each coincidence count are considered to follow a Poisson distribution and for each coincidence count we generate plenty of random numbers with a Poisson distribution. With these random numbers we obtain different density matrices and with these density matrices we can calculate the standard deviations of the fidelity and concurrence of the simulated ground state and two-spin correlation. In our experiment, the fidelity is limited by the imperfection of the preparation of the initial state and the accuracies of the optical elements such as wave plates.

**Table 1:** The dimensionless magnetic field strength *β*_*z*_ (here *β*_*x*_ = 0.15), the angles of the HWPs *α* and *ϑ*, the measured concurrence ${\mathscr{C}}$, the two-spin correlation $\langle {\sigma }_{z}^{1}{\sigma }_{z}^{2}\rangle$ and fidelity of the simulated ground state compared to the theoretical predictions ${\mathcal F}$.

*β*_*z*_	*α*	*ϑ*	${\bf{C}}$	$\langle {{\boldsymbol{\sigma }}}_{{\boldsymbol{z}}}^{{\bf{1}}}{{\boldsymbol{\sigma }}}_{{\boldsymbol{z}}}^{{\bf{2}}}\rangle$	${\boldsymbol{ {\mathcal F} }}$
−3.0	−3.02°	−0.11°	0.027(16)	0.996(2)	0.996(1)
−2.5	−4.02°	−0.20°	0.051(20)	0.984(3)	0.997(1)
−2.0	−5.96°	−0.46°	0.042(13)	0.965(4)	0.996(3)
−1.5	−11.21°	−1.84°	0.020(16)	0.885(9)	0.994(4)
−1.0	−32.59°	−20.04°	0.608(13)	−0.315(20)	0.970(3)
−0.5	−43.12°	−37.33°	0.909(28)	−0.905(8)	0.965(15)
0.0	−45°	−39.08°	0.861(38)	−0.929(9)	0.939(18)
0.5	−46.88°	−37.33°	0.930(10)	−0.931(7)	0.969(8)
1.0	−57.41°	−20.04°	0.604(13)	−0.380(15)	0.956(3)
1.5	−78.79°	−1.84°	0.017(10)	0.817(11)	0.984(3)
2.0	−84.04°	−0.46°	0.015(16)	0.954(4)	0.991(3)
2.5	−85.98°	−0.20°	0.033(15)	0.972(5)	0.994(2)
3.0	−86.98°	−0.11°	0.062(19)	0.987(4)	0.997(2)

Error bars indicate the statistical uncertainty and are estimated with Monte Carlo simulation.

**Figure 3:**
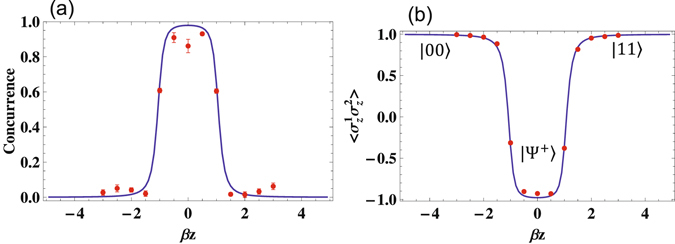
(**a**) Measured concurrence of the simulated ground state of the two-spin Ising model v.s. the dimensionless longitudinal field strength *β*_*z*_ and fixed transverse filed strength *β*_*x*_ = 0.15, compared to the theoretical prediction (solid line). (**b**) Measured two-spin correlation $\langle {\sigma }_{z}^{1}{\sigma }_{z}^{2}\rangle$ as a function of *β*_*z*_ compared to the theoretical prediction. Error bars indicate the statistical uncertainty and are estimated with Monte Carlo simulation. Some of them are smaller than portrayed by the symbols.

In this paper, we use the same figures of merit as used in ref. 31. We use the concurrence ${\mathscr{C}}$ as the order parameter, which is related to the entanglement of formation. The concurrence as an entanglement monotone is defined for a mixed two-qubit state *ρ*

7 $${\mathscr{C}}(\rho )\,=\,{\rm{\max }}\,\{0,{\zeta }_{1}-{\zeta }_{2}-{\zeta }_{3}-{\zeta }_{4}\},$$

where *ζ*_*i*_ is the eigenvalue, in decreasing order, of the Hermitian matrix $R=\sqrt{\sqrt{\rho }\tilde{\rho }\sqrt{\rho }}$ with $\tilde{\rho }=({\sigma }_{y}^{1}{\sigma }_{y}^{2}){\rho }^{\ast }({\sigma }_{y}^{1}{\sigma }_{y}^{2})$. Figure 3a shows the measured concurrences as 13 individual points with the parameters {*β*_*x*_, *β*_*z*_} = {0.15, 0}, {0.15, ±0.5}, {0.15, ±1}, {0.15, ±1.5}, {0.15, ±2}, {0.15, ±2.5}, {0.15, ±3}, which agree well with the theoretical predictions represented in solid line. From the concurrence of the simulated ground state, we can clearly observe the expected ground state transportation occurring near the critical points *β*_*z*_ = ±1. The simulated ground state shows entanglement property for −1 < *β*_*z*_ < 1. With |*β*_*z*_| increasing, the simulated ground state changes from the entangled, antiferromagnetic low-field state to the separated, ferromagnetic high-field state. The measured concurrence dropped down to 0 quickly with |*β*_*z*_| increasing.

Furthermore, we use the two-spin correlation

8 $$\langle {\sigma }_{z}^{1}{\sigma }_{z}^{2}\rangle ={\rm{Tr}}(\rho {\sigma }_{z}^{1}{\sigma }_{z}^{2})$$

as another order parameter. As theoretical predictions, the ground state with $\langle {\sigma }_{z}^{1}{\sigma }_{z}^{2}\rangle =1$ is ferromagnetically ordered for high magnetic fields and it is transmitted to the antiferromagnetic state with $\langle {\sigma }_{z}^{1}{\sigma }_{z}^{2}\rangle =-\,1$ for low magnetic fields at the critical points *β*_*z*_ = ±1. The measure two-spin correlations shown in Fig. 3b agree with the theoretical predictions. The simulated ground state of the two-spin Ising model changes from the ferromagnetically ordered high-field states to the entangled anti-ferromagnetic low-field states via tuning the setting angles *ϑ*/2 and *α*/2 of the HWPs to meet the critical points *β*_*z*_ = ±1.

## Discussion

Much attention has been attracted by the development of a quantum computer relates to its application to simulating dynamics of another complex system whose properties cannot easily be computed with classical computers. A controllable system can be used for reproducing the dynamics and the quantum state of another system of study. This idea forms the foundation of quantum simulation. In this paper we experimentally generate the ground state of the two-spin Ising model Hamiltonian with two external magnetic fields. The implemented quantum processor consists of generation of photonic entangled state with controllable coefficients and local operations. We tune the ratio of magnetic fields and coupling strength by changing local parameters to observe quantum state transportation: the ground state is transmitted from the ferromagnetically ordered high-field states to the entangled anti-ferromagnetic low-field states. This simulation of the Ising model with linear quantum optics might open the door to the future studies which connect quantum information and condensed-matter physics. To extend the method in our experiment to simulate the ground state of Ising-type *N* spins the major challenge is to generate systems with more photonic qubits. Recently the experimental generation of the 10-photon entangled states has been reported^36^. Thus our method can be extended to simulate the ground state of Ising-type *N* spins (*N* ≤ 10) under the current technologies.
